# LncRNAs Are Key Regulators of Transcription Factor-Mediated Endothelial Stress Responses

**DOI:** 10.3390/ijms25179726

**Published:** 2024-09-08

**Authors:** Frederike Lam, Matthias S. Leisegang, Ralf P. Brandes

**Affiliations:** 1 Goethe University, Institute for Cardiovascular Physiology, Frankfurt, Germany; 2 German Center of Cardiovascular Research (DZHK), Partner Site RheinMain, Frankfurt, Germany

**Keywords:** LncRNA, transcription factor, endothelial stress response, hypoxia, laminar shear stress, transcription control

## Abstract

The functional role of long noncoding RNAs in the endothelium is highly diverse. Among their many functions, regulation of transcription factor activity and abundance is one of the most relevant. This review summarizes the recent progress in the research on the lncRNA–transcription factor axes and their implications for the vascular endothelium under physiological and pathological conditions. The focus is on transcription factors critical for the endothelial response to external stressors, such as hypoxia, inflammation, and shear stress, and their lncRNA interactors. These regulatory interactions will be exemplified by a selected number of lncRNAs that have been identified in the endothelium under physiological and pathological conditions that are influencing the activity or protein stability of important transcription factors. Thus, lncRNAs can add a layer of cell type-specific function to transcription factors. Understanding the interaction of lncRNAs with transcription factors will contribute to elucidating cardiovascular disease pathologies and the development of novel therapeutic approaches.

## 1. Introduction

**Figure 1:**
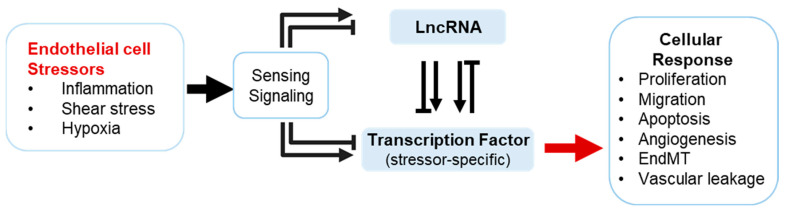
The endothelial lncRNA–transcription factor axis. Endothelial cells are subjected to different stressors, including inflammation, hypoxia, and shear stress. Upon sensing and initial signaling, transcription factors or lncRNAs act on each other’s function, activity, or abundance in order to facilitate an adequate cellular response to external stimuli. These responses include changes in proliferation, migration, apoptosis, angiogenesis, endothelial-to-mesenchymal transition (EndMT), and vascular barrier function.

The endothelium, the cellular monolayer lining the interior of blood vessels, plays a pivotal role in maintaining vascular homeostasis [1]. Its functional integrity is crucial for processes such as angiogenesis, vascular permeability and vascular tone, coagulation, immune cell trafficking, and inflammation, as well as the regulation of the oxygen and nutrient supply [2,3]. The regulation of endothelial gene expression is therefore of great importance in cardiovascular physiology and pathology. Recent advances have illuminated the intricate complexity of this regulation, revealing that noncoding RNAs, particularly long noncoding RNAs (lncRNAs), are key players in modulating endothelial gene expression (Figure 1) [4,5,6].

**Figure 2:**
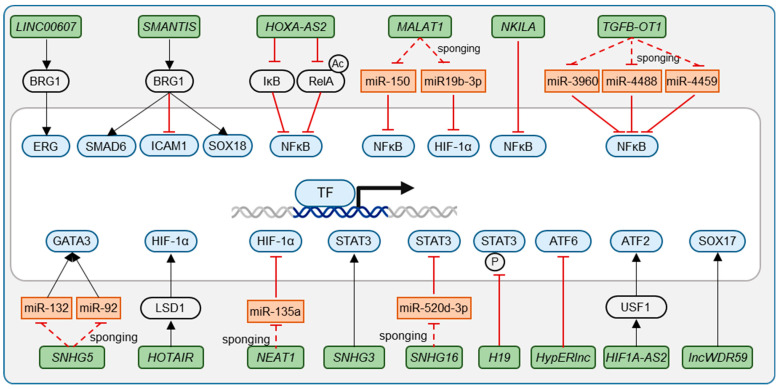
LncRNAs that influence TF activity. LncRNAs can promote or repress TF activity directly or by sponging of miRNAs.

**Figure 3:**
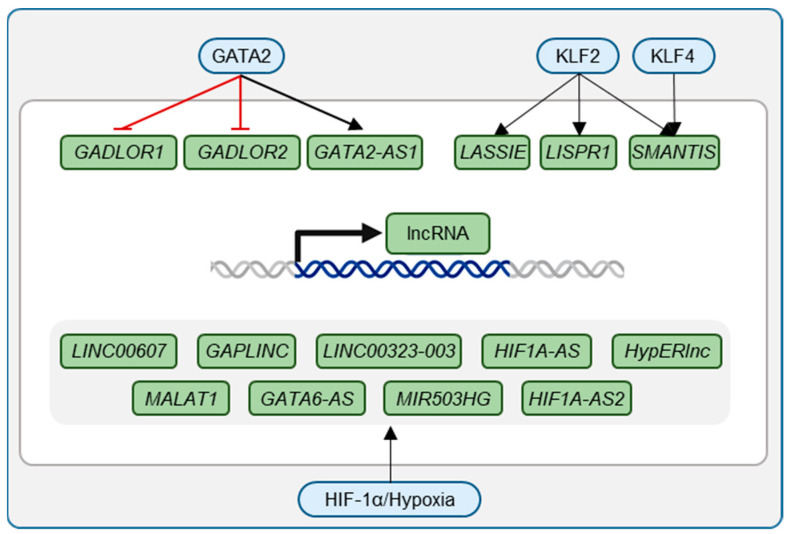
TFs that influence LncRNAs. TFs can promote or repress lncRNA expression.

**Figure 4:**
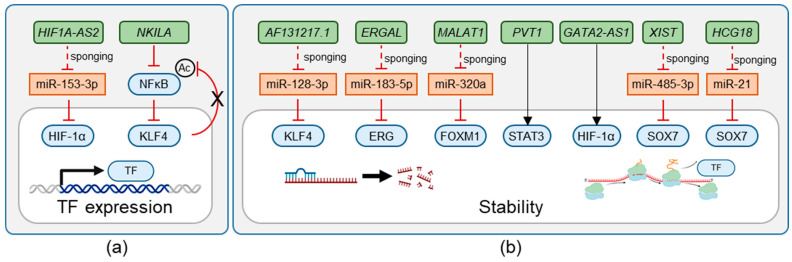
LncRNAs influence TF abundance. LncRNAs can promote or repress TF abundance by acting on their transcription (**a**) or mRNA stability, translation, or protein stability (**b**).

LncRNAs are a diverse class of RNA molecules exceeding 200 nucleotides in length that, despite lacking protein-coding potential, exhibit profound regulatory capabilities [4]. LncRNAs influence gene expression through various mechanisms, including chromatin remodeling, transcriptional regulation, and post-transcriptional modification (Figure 2, Figure 3 and Figure 4) [5]. In the context of endothelial biology, lncRNAs have emerged as critical modulators orchestrating the expression of genes essential for endothelial cell function in healthy endothelium or in cardiovascular diseases (CVDs) [6,7,8,9]. LncRNAs have also been linked to the main stressors underlying CVDs: inflammation, shear stress, and hypoxia [9,10]. The predominant localization of lncRNAs within the nucleus indicates their involvement in transcriptional regulation [4,5]. The interaction with transcription factors (TFs) is, among other mechanisms, a major regulatory mechanism of lncRNAs [4,5,9,10].

TFs are proteins that bind to specific DNA motifs to regulate the transcription of genetic information from DNA to mRNA. They are the pivotal factors in controlling gene expression [11]. As most lncRNA expression is cell-type-specific, the interaction between lncRNAs and TFs adds a complex layer to the cell-type-specific regulation of endothelial gene expression, influencing a variety of physiological and pathological processes [5,9]. The endothelium can be defined by a set of (endothelial-) specific TFs that are crucial for adequate cellular responses to external stimuli or the overall maintenance of endothelial homeostasis. Such TFs belong, for example, to the Sox, Kruppel-like, Forkhead Box (FOX), Erythroblast Transformation Specific (ETS), and GATA families [12,13]. Interestingly, Melé et al. identified binding motifs for many endothelial TFs to be conserved in the promoter region of long intergenic noncoding RNAs (lincRNAs) [14]. Among other motifs, conserved motifs for GATA2 and GATA3, members of the FOX TFs, and STAT1 and STAT3 were identified. This promotes the importance of the functional relation between lncRNAs and TFs, as TF binding motifs in intergenic regions were subjected to evolutionary conservation [14]. Subsequently, the selection of TFs reported in this review was based on those shown to be crucial for endothelial function (ERG, KLF2, KLF4, NF-κB, HIF-1α, SMAD6, SOX7, SOX17, SOX18) and to the TFs with conserved binding motifs in the promoters of lincRNAs (GATA2, GATA3, FOXM1, STAT1, STAT3), with reported lncRNA interactions in the endothelium [12,13,14].

This review aims to elucidate the mechanisms by which lncRNAs control endothelial gene expression through their interactions with TFs. We explore the multifaceted roles of lncRNAs in endothelial function, detailing how their interactions with TFs contribute to the fine-tuning of gene expression. By summarizing current knowledge and highlighting recent discoveries, this review provides a comprehensive understanding of the lncRNA–transcription factor axis in endothelial biology, offering insights into potential therapeutic targets for CVDs.

## 2. Key Endothelial Transcription Factors and Their Regulatory lncRNA Partners

In the endothelium, a number of TFs are known to regulate the cellular response to stimuli, including hypoxia, shear or oxidative stress, and inflammation [12]. Understanding the role of these endothelial TFs provides insight into the molecular mechanisms governing endothelial function. Here, we highlight some of the most common and pivotal endothelial TFs and their functional association with lncRNAs.

### 2.1. Ets Family Transcription Factors

The ETS TF family is one of the most prominent protein families, with a TF recognition motif present in virtually every gene promoter of endothelial signature genes [13,15]. The individual ETS TF family members execute specific functions, which are carried out by binding to their DNA recognition motif with the consensus sequence: 5′-RSAGGAAG-3′ [16]. ERG (ETS Transcription Factor ERG) is the most prominent ETS family member, with high expression in mature human ECs [17]. Among the gene programs activated by ERG are blood vessel morphogenesis and angiogenesis in response to Vascular Endothelial Growth Factor (VEGF) stimulation [18]. ERG carries out both a repressive and activating function to regulate gene expression in response to different endothelial stimuli [18]. Until now, ERG has not been deeply linked to the induction of lncRNAs.

ERG’s function and expression are controlled by the lncRNAs *Long intergenic noncoding RNA 607* (*LINC00607*) and *ERG-associated lncRNA* (*ERGAL*). The endothelial-enriched *LINC00607* maintains accessible chromatin states at ERG target genes, primarily through its interaction with the chromatin remodeler Brahma-related gene-1 (BRG1) (Figure 2) [19]. Therefore, *LINC00607* is essential for angiogenesis and proper endothelial cell function, making it a potential target for therapeutic interventions in vascular diseases [19]. The Dengue virus-induced lncRNA *ERGAL* safeguards the endothelial barrier integrity by acting as a competing endogenous RNA (ceRNA) for miR-183-5p. MiR-183-5p would otherwise induce degradation of ERG and subsequently decrease expression of VE-cadherin and claudin-5 (Figure 4) [20]. *ERGAL* is needed for maintaining endothelial barrier function during DENV infection [21]. These examples show the impact lncRNAs can have on ERG-mediated endothelial homeostasis and vascular integrity.

### 2.2. Krüppel-like Factors—KLF2 and KLF4

Krüppel-like factors 2 and 4 (KLF2 and KLF4) are C2H2-type zinc-finger TFs that are described as atheroprotective factors [22]. These KLFs are essential for the maintenance of endothelial homeostasis in response to shear stress [23,24]. Downstream target genes of KLF2 and KLF4 include NOS3 (endothelial nitric oxide synthase), thrombomodulin, and Heme Oxygenase 1 (HO1) [23,25,26]. KLF expression is in turn activated by myocyte enhancer factor-2 (MEF2) [27]. KLFs are not only important for direct transcription factor activity but also for facilitating chromatin accessibility in vasculo-protective genes. Here, KLF4 recruits not only the SWI-SNF chromatin remodeling protein BRG1 but also binds at enhancer loops in primary human pulmonary artery endothelial cells [28]. 

Both KLF2 and KLF4 have been functionally linked to lncRNAs. The lncRNA *AF131217.1* plays a potential role in inhibiting inflammation by regulating the expression of KLF4. *AF131217.1* is upregulated in HUVECs exposed to laminar shear stress. *AF131217.1* acts as a sponge for miR-128-3p, thereby preventing miR-128-3p from binding to and downregulating *KLF4* mRNA. This leads to increased *KLF4* expression, which has anti-inflammatory effects on endothelial cells [29]. The KLF2-induced lncRNA *LASSIE* was identified as a critical regulator of endothelial cell function under shear stress conditions, mediating proper mechanosensing and junctional stability responses downstream of KLF2 [30]. A lncRNA that has been shown to be induced by KLF2 and KLF4 is *MANTIS* (now named *SMARCA4 Interacting SWI/SNF Chromatin Remodeling Complex Scaffold LncRNA* (*SMANTIS*)). In response to laminar flow and statin treatment, *SMANTIS* is upregulated by KLF2 and KLF4, which bind to the promoter of *SMANTIS*. *SMANTIS*, in turn, limits the expression of Intercellular Adhesion Molecule 1 (ICAM-1) by preventing the binding of the chromatin remodeling factor BRG1 to the *ICAM-1* promoter, thereby reducing monocyte adhesion to endothelial cells [31].

Another KLF2-dependent lncRNA is *Long intergenic noncoding RNA antisense to S1PR1* (*LISPR1*), which is needed for migration and sprouting. In HUVECs, the knockdown of KLF2 decreases *LISPR1* expression since KLF2 binds the promoter of *LISPR1*, which is further increased under laminar shear stress [32]. The lncRNA *NF-κB-interacting lncRNA (NKILA)* regulates KLF4 expression through interaction with NF-κB [33]. These lncRNAs play a pivotal role in reducing inflammatory signaling in response to laminar shear stress by the regulation of KLF2 and KLF4, thereby maintaining vascular homeostasis.

### 2.3. Nuclear Factor-κB (NF-κB)

The NF-κB family of TFs is central to the regulation of inflammatory responses in endothelial cells [34]. NF-κB can be activated by various stimuli, including cytokines, shear stress, and oxidative stress, leading to the transcription of genes involved in inflammation, immune responses, and cell survival [34,35]. Chronic activation of NF-κB is associated with endothelial dysfunction and the development of CVDs, such as atherosclerosis [36].

The lncRNA *HOXA*-*AS2* significantly represses endothelial inflammation by controlling IκBα degradation and RelA acetylation. *HOXA-AS2* expression correlates with carotid artery atherosclerosis by inhibiting NF-κB signaling. The results of Zhu et. al. establish a negative feedback loop that inversely activates *HOXA-AS2* transcription elongation. *HOXA-AS2* was shown to be a critical repressor of endothelial inflammation by balancing NF-κB signaling [37]. Other lncRNAs have been shown to indirectly influence NF-κB through miRNA sponging as competing endogenous RNAs (ceRNAs). An example is *MALAT1* interacting with miR-150. In pulmonary arterial ECs, miR-150 suppresses endoplasmic reticulum stress in response to LPS (lipopolysaccharide) treatment and in septic mice. *MALAT1* sponges miR-150, driving NF-κB signaling [38]. Another example is the lncRNA *TGFB2-OT1* that is induced by LPS and oxLDL (oxidized low-density lipoprotein) stimulation. *TGFB2-OT1* itself induces autophagy and inflammation by sequestering the miRNAs miR-3960, miR-4488, and miR-4459. In the absence of *TGFB2-OT1*, these miRNAs repress the expression of CERS1, NAT8L, ATG13, and LARP, which induce autophagy and drive inflammation [39]. In healthy endothelium, *NKILA* represses NF-κB activity, thus promoting the expression of KLF4. In an inflammatory environment, *NKILA* dissociates from NF-κB, enabling nuclear translocation and repression of KLF4 expression [33]. These examples give further indication of the involvement of lncRNAs in the fine-tuned inflammatory response of ECs through the NF-κB signaling pathway.

### 2.4. GATA Transcription Factors

The GATA transcription factor family consists of six members (GATA1-6). These zinc finger TFs can be divided into two subfamilies. GATA1, GATA2, and GATA3 form the “hematopoietic group”, with strong expression in hematopoietic stem cells. GATA4, GATA5, and GATA6 make up the “cardiac group”, with high expression in the heart and gut [12,40]. GATA TFs bind the DNA motif 5′-WGATAR-3′ [16]. In endothelial cells, GATA2, GATA3, and GATA6 are the most highly expressed compared with the other members of the TF family [41]. Particularly, GATA2 and GATA3 are crucial for endothelial cell function and vascular integrity [12]. GATA2 is expressed in endothelial cells and regulates genes involved in vascular development and hemodynamic response [40]. It also plays a role in maintaining endothelial cell survival and function under stress conditions [40]. GATA3, while more extensively studied in the context of immune cell differentiation, has emerging roles in endothelial biology, such as the regulation of Tie2 expression [42]. GATA6 is crucial for endothelial cell survival and angiogenesis [43].

GATA2 and GATA3 transcriptional functions have been linked to lncRNAs, whereas GATA6 has not. Fiedler et. al. identified two hypoxia-sensitive lncRNAs, *LINC00323-003* and *MIR503HG*, which are essential for maintaining vascular homeostasis and endothelial cell biology through GATA2. Decreased expression of *LINC00323-003* and *MIR503HG* initiates a series of antiangiogenic events by repressing GATA2 [44]. *LINC00323-003* potentially interacts with the translation initiation factor eIF4A3 to regulate GATA2 translation [44], while lncRNA *MIR503HG*, which is repressed under EndMT [45], influences the expression of the adjacent miRNA miR-424 [44]. Another set of lncRNAs that are in turn regulated by GATA2 are the *GATA-Dependent Long noncoding RNA 1* and *2* (*GADLOR1* and *2*). In healthy cardiac ECs, expression of *GADLOR1/2* is repressed by GATA2. Upon mechanical overload, GATA2 expression is decreased, resulting in the induction of *GADLOR1/2*. The lncRNAs are taken up by cardiomyocytes, where they interfere with the activation of p38 and Akt [46]. 

A potential ceRNA immune-regulatory network was identified in the acute and febrile systemic vasculitis called Kawasaki disease (KD) involving GATA3. Co-expression studies and predicted regulatory networks indicated that *SNHG5*-mediated repression of GATA3 through sponging the miRNAs miR-132 and miR-92 might have crucial roles in the pathology of KD through regulating inflammation [47]. Although GATA6 activity was not reported to be modulated by lncRNAs, the *GATA6* locus includes the antisense lncRNA *GATA6-AS*, which is important for the epigenetic gene regulation of proangiogenic and endothelial-to-mesenchymal transition (EndMT)-related gene programs [48]. These examples show the importance of the lncRNA-GATA axis for the maintenance of vascular homeostasis and the progression of CVDs.

### 2.5. Hypoxia-Inducible Factor (HIF)

HIF-1 and HIF-2 are central TFs in the response of endothelial cells to hypoxia [49,50]. In a normoxic environment, HIF-1α is degraded by the proteasome. Under low oxygen conditions, HIF-1α stabilizes and translocates to the nucleus, where it dimerizes with HIF-1β and activates the transcription of genes involved in angiogenesis, metabolism, and cell survival [51]. As a dimer, the basic helix–loop–helix TF factors bind the hypoxia response elements (HREs) with the core consensus sequence 5′-RCGTG-3′ [16]. This mechanism is crucial for the adaptation of endothelial cells to hypoxic environments, such as those found in ischemic tissues [51,52]. 

The *HIF1A* locus also includes antisense lncRNAs. Under hypoxic conditions, some of these lncRNAs repress HIF-1α expression in *cis* as part of a negative feedback loop through repressing transcriptional elongation and deposition of H3K4me3 [49]. In HUVEC, *HIF1A-AS2* also serves a *trans*-acting function, inducing proangiogenic signaling through the upregulation of HIF-1α by sponging miR-153-3p [53]. In addition, *HIF1A-AS2* aggravates inflammation in atherosclerosis by inducing Activating Transcription Factor 2 (ATF2) [54]. Under hypoxic conditions, it recruits the transcription factor Upstream Stimulatory Factor 1 (USF1) to the *ATF2* locus, activating its transcription and driving atherosclerotic inflammation [54]. This effect was reversed by silencing *HIF1A-AS2* [54]. The antiangiogenic lncRNA *HIF1A-AS1* suppresses gene expression of *EPHA2* through recruitment of the human silencing hub complex (HUSH) by forming RNA-DNA triplexes with target genes in ECs [55]. With increased expression of *HIF1A-AS1* in thoracoabdominal aortic aneurysms, it is also relevant in the pathogenesis of CVDs [56]. Additionally, a large *HIF1A-AS* was found to be induced by hypoxia, HIF-1α and HIF-2α, which in turn was able to repress *HIF-1*α mRNA expression [49].

HIF-1α function is also regulated by other lncRNAs. An example is *HOTAIR*, which facilitates the association of LSD1 and HIF-1α to induce VEGF transcription in retina ECs under high glucose conditions in diabetic retinopathy [57]. *MALAT1* can improve the HIF-1α-mediated hypoxic response of ECs by sponging miR-19b-3p [58]. *NEAT1* functions as a negative regulator of the blood–brain barrier (BBB) by binding to miR-135a. If not bound to *NEAT1*, miR-135a reduces BBB permeability by targeting HIF-1α [59]. Also, lncRNA *SNHG1* (*small nucleolar RNA host gene 1*) regulates the HIF-1α/VEGF signaling pathway. It offers neuroprotection in ischemic stroke by acting as a ceRNA for miR-18a, promoting brain microvascular endothelial cell proliferation [60]. In myocardial ischemia/reperfusion injury, *SNHG1* serves a similar protective role by acting as a ceRNA for miR-140-3p, thereby preventing hypoxia–reoxygenation-induced vascular endothelial cell injury [61]. In both instances, *SNHG1* promoted the activity of the HIF-1α/VEGF signaling pathway [60,61]. *GATA2-AS1* is an important lncRNA for adequate endothelial response to chronic and acute hypoxia through HIF-1α. It is essential for increasing HIF-1α protein levels during acute hypoxia but plays a minor role during chronic hypoxia. *GATA2-AS1* affects HIF-1α post-transcriptionally and supports metabolic reprogramming towards glycolysis while maintaining mitochondrial function [62]. 

There are many hypoxia- and HIF-1α-inducible lncRNAs [19,44,48,49,63,64,65]. Among them, *GAPLINC*, induced under hypoxic conditions, promotes the angiogenic response in HUVECs by in turn potentially promoting Vascular Endothelial Growth Factor Receptors (VEGFR) and delta-like canonical notch ligand 4 (DLL4) (Figure 3) [64]. Also, *H19*, *Meg9*, *Malat1*, and *MIR22HG* were induced by hypoxia in HUVEC and in mouse hindlimb ischemia [66]. Another example is the *Hypoxia-Induced Endoplasmic Reticulum Stress Regulating lncRNA* (*HypERlnc*). Loss of *HypERlnc* in pericytes and cardiomyocytes leads to enhanced ER stress response through a feedback loop with the transcription factors CBF/NF-Y/YY1 and ATF6 [67]. As a consequence, pericyte recruitment to human microvascular endothelial cells (HMVEC) is reduced, causing vascular leakage [67]. Reduced expression of *HypERlnc* was observed in samples from failing hearts [67].

In addition to the mentioned lncRNA-HIF-1α interactions in the endothelium, there are several reports on the relevance of the lncRNA-HIF-1α axis for cancer development and progression. Especially, the induction of the Vascular Endothelial Growth Factor (VEGF) in malignant tissue by HIF-1α through lncRNAs is a major contributor to tumor angiogenesis [68,69,70,71]. As a crucial endothelial TF, HIF-1α activity, stability, and abundance are influenced by lncRNAs in various disease contexts, making lncRNAs suitable therapeutic targets in ischemic diseases.

### 2.6. Sma- and Mad-Related Proteins (SMADs) and Forkhead Box (FOX) Proteins

Sma and mothers against decapentaplegic (mad) proteins (SMADs) proteins are the transcriptional effectors of the TGFβ superfamily [72]. As such, they are involved in the inflammatory response and the endothelial-to-mesenchymal transition (EndMT) of ECs [73,74]. The SMAD TF family can be separated into three groups: the first group is the receptor-associated SMADs, including R-SMADs and SMAD1/2/3/5/8, the second group consists of the common SMAD, which is SMAD4, and the third group is the inhibitory SMADs, called I-SMADs, consisting of SMAD6/7 [74,75]. Even though many lncRNAs are reported to influence the TGFβ/SMAD pathway, most of them influence the activation of the TGFβ-receptors. Examples include the lncRNAs *SENCR* [76], *ANRIL* [77], *NEAT1* [78], and *ZEB1-AS1* [79]. An example of the regulation of SMADs is the KLF2/4-induced lncRNA *SMANTIS*. *SMANTIS* guides the chromatin remodeling protein BRG1 to activate the expression of the I-SMAD SMAD6 [80]. 

Similarly poorly analyzed are FOXOs and their potential lncRNA interaction partners and targets in endothelial cells. Among the different functions of Forkhead box protein M1 (FOXM1) is the transcriptional regulation of many cell cycle G2/M phase-specific genes [81]. A lncRNA linked to the regulation of FOXM1 in the context of proliferation is *MALAT1*. In HUVEC, *MALAT1* was shown to promote the stability of FOXM1 by sponging miR-320a [82].

### 2.7. Signal Transducer and Activator of Transcription (STAT) Proteins

The signal transducer and activator of transcription factor 3 (STAT3) is a prominent member of the STAT family that is involved in mediating responses to cytokines and growth factors, such as VEGF, in endothelial cells [83,84]. It plays a role in promoting cell survival, proliferation, and angiogenesis. Dysregulation of STAT3 signaling is linked to various pathological conditions, including chronic inflammation and tumor angiogenesis [85].

Through direct binding, the lncRNA *PVT1* protects STAT3 from proteasomal degradation, thereby activating the STAT3 signaling pathway and increasing VEGFA expression to stimulate angiogenesis. This *PVT1*-STAT3-VEGFA axis, verified in gastric cancer specimens, forms a positive feedback loop that correlates with enhanced tumor angiogenesis and worsened overall survival [86]. In microvascular brain ECs, in an intracerebral hemorrhage model, the lncRNA *SNHG3* expression is induced, contributing to the dysfunction of cerebral microvascular cells by activating the TWEAK/Fn14/STAT3 pathway. By increasing the expression of TNF-related weak inducer of apoptosis (TWEAK) and its receptor Fn14, STAT3 activation and enhanced secretion of MMP-2 and MMP-9 further worsened the dysfunction of the blood–brain barrier [87]. In hemangioma ECs, *SNHG16* positively modulated STAT3 expression by sequestering miR-520d-3p. By acting as a ceRNA, *SNHG16* drives the proliferation, migration, and invasion of hemangioma endothelial cells [88]. The lncRNA *H19* is repressed during aging and controls endothelial cell senescence, proliferation, inflammatory activation, and angiogenic sprouting by suppressing STAT3 phosphorylation and thus its activation and target gene expression [89].

### 2.8. SRY (Sex Determining Region Y)-Related HMG Box of DNA Binding Proteins—SOX

The SRY (Sex Determining Region Y)-related HMG box of DNA binding protein (SOX) TF family plays an important role not only in male sex determination but also in vascular development and disease. The SOX TFs recognize and bind the DNA consensus motif 5′-WWCAAWG-3′ [16]. The SOX TF family consists of 20 genes classified into eight groups (A-H) [90]. The SOX-F group, consisting of SOX7, 17, and 18, is crucial for endothelial differentiation, angiogenesis, and vasculogenesis [91]. Interestingly, SOX2 was shown to mediate endothelial–mesenchymal transitions (EndMT) [91]. 

LncRNAs have been functionally linked to SOX TFs in the context of endothelial dysfunction. SOX7 function is regulated by two lncRNAs for the maintenance of endothelial barrier integrity. In human brain microvascular ECs, the hypoxia-induced lncRNA *XIST* was shown to promote SOX7-mediated angiogenesis. *XIST* acts here as a ceRNA for miR-485-3p, which otherwise decreases SOX7 abundance by binding SOX7 mRNA. Upregulation of *XIST* thereby increases SOX7 levels, resulting in increased activation of the VEGF signaling pathway (Figure 4). The induction of VEGF signaling results in angiogenesis and a reduction in the vascular barrier. Therefore, *XIST* was proposed as a molecular target after ischemic stroke [65]. Another example is the lncRNA *Hickson compact group 18* (*HCG18*). *HCG18* similarly acts as a ceRNA for miR-21. Like miR-485-3p, miR-21 regulates SOX7 abundance. In the presence of ambient particulate matter with an aerodynamic diameter < 2.5 μm (PM2.5), *HCG18* safeguards the integrity of the vascular barrier, enabling SOX7-mediated VE-cadherin induction. PM2.5 itself downregulates *HCG18*, aggravating endothelial barrier breakdown (Figure 4) [92].

SOX17 activity has also been linked to a lncRNA. Functionally, Sox17 limits EC proliferation and reduces micronucleic DNA damage. In murine aortic ECs, the lncRNA *WD Repeated Domain 59* (*lncWDR59*) prevents oxidative stress-induced DNA damage by upregulating SOX17 expression through Notch1, thereby promoting β-catenin activity. *lncWDR59* is inhibited by miR-103, a miRNA upregulated in hyperlipidemia and oxLDL. Therefore, the *lncWDR59*-miR-103 axis might be an important factor in ECs reprogramming toward a maladapted phenotype under disturbed flow [93]. Like the Nuclear receptor subfamily 2 group F member 2 (NR2F2, known as COUP-TFII) and SMAD6, the TF SOX18 is also functionally dependent on the lncRNA *SMANTIS*, which secures SOX18 expression through BRG1 [80]. Recent studies could show a clinical relevance of the *SMANTIS*-SOX18 axis in chronic kidney disease (CKD): *SMANTIS* expression is decreased in CKD and in a protein-bound uremic-toxin-induced HUVECs injury model. This resulted in decreased SOX18 expression and enhanced p38 MAPK and p65 NF-κB signaling, further driving disease progression. This suggests that *SMANTIS* is a potential target in treating CKD [94]. Additionally, it has been discovered that Dioscin, a plant-derived saponin that has been reported to exert positive effects for the treatment of coronary artery disease (CAD), functions through the induction of *SMANTIS*. The elevation of *SMANTIS* expression, and subsequently SOX18, SMAD6, and COUP-TFII, results in the cardio-protective effect of Dioscin [95]. Taken together, these examples show how important the lncRNA regulation of SOX TFs is for endothelial barrier integrity and homeostasis, as well as in the treatment of CVDs, such as CAD or CKD. 

## 3. Conclusions

LncRNAs have emerged as pivotal regulators of gene expression, particularly through their interactions with TFs in the endothelium. LncRNAs play crucial roles in maintaining endothelial cell function under both physiological and pathological conditions. By modulating the activity, abundance, and stability of key TFs, such as HIF-1α, SOX, ETS, and GATA, lncRNAs orchestrate complex cellular responses to various stimuli, including hypoxia, inflammation, and shear stress (Table 1).

However, there are also many individual cardiovascular important lncRNAs regulated by or regulating TFs not matching the TF classes presented here, among them are *TERMINATOR* [96], *SARRAH* [97], *PCAT19* [98], or *MEG3* [99], implying that more lncRNA-TF axes exist. Additionally, through R-loop or RNA-DNA triplex formation, lncRNAs may affect or facilitate the binding of TFs to DNA to regulate cardiovascular-specific gene programs [100]. It should also be mentioned that modulation of topoisomerase activity can affect lncRNA and transcription factor expression levels (such as GATA4, FOXA1, FOXA3, and IRF4) and therefore potentially regulate cardiovascular gene programs [101].

Under physiological conditions, lncRNAs contribute to the fine-tuning of vascular homeostasis, promoting adaptive responses that ensure proper blood flow and nutrient delivery. They regulate processes such as angiogenesis, vascular permeability, and metabolic adaptation, which are essential for maintaining endothelial integrity and function. For instance, the lncRNA *GATA2-AS1* is crucial for the induction of HIF-1α during acute hypoxia, facilitating metabolic reprogramming towards glycolysis and supporting endothelial cell survival and function [62]. In pathological states, such as cardiovascular diseases, diabetes, and cancer, the dysregulation of lncRNA-TF interactions can lead to aberrant endothelial responses, contributing to disease progression. lncRNAs can either exacerbate or mitigate pathological conditions by influencing TF activity. For example, altered expression of lncRNAs may disrupt the balance between proangiogenic and antiangiogenic signals, leading to abnormal blood vessel formation and vascular dysfunction.

The investigation of lncRNA promoters and TF motifs within them will additionally provide insights into tissue-specific lncRNA-TF pairs [14]. The main difference between intergenic lncRNA (lincRNA)- and divergent lncRNA promoters is the absence or presence of overlapping TF motifs [102]. LincRNAs tend to have shorter and less overlapping motifs, responsible for lower expression levels with higher tissue specificity. The majority of tissue-specific TFs bind to short, less complicated DNA motifs found in lincRNA promoters. Overlapping DNA motifs in promoters of protein-coding genes are subjected to evolutionary conservation. Pairs of lincRNAs and tissue-specific TFs are likely to be less evolutionarily conserved due to their fewer overlapping motifs [14,102].

Understanding the intricate relationships between lncRNAs and transcription factors in the endothelium opens new possibilities for therapeutic interventions. Targeting specific lncRNAs or their interactions with TFs holds promise for developing novel treatments for a range of vascular diseases and improving disease outcomes [9,103]. As research on lncRNAs progresses, the potential to modulate lncRNAs for precise fine-tuning of endothelial function represents a significant step forward in vascular biology and medicine.

## Figures and Tables

**Table 1:** Summary of crucial endothelial lncRNAs in control of TF activity and abundance.

Transcription Factor	lncRNA	Mechanism	Relevance	Source
ERG	*LINC00607*	BRG1-mediated ERG DNA motif accessibility		[19]
	*ERGAL*	ceRNA for miR-183-5p, preventing degradation of ERG	Dengue Virus infection	[21]
KLF2/KLF4	*SMANTIS*	Scaffold for chromatin remodeling protein BRG1	Laminar shear stress, statin treatment	[31]
KLF2	*LASSIE*	Regulation of barrier function by connecting adherens junctions to the cytoskeleton	Laminar shear stress	[30]
	*LISPR1*	Needed for migration and sprouting; upregulated under laminar flow and statins	Reduced in patients with COPD, CTEPH, and IPAH	[32]
KLF4	*AF131217.1*	Promoting anti-inflammatory phenotype. ceRNA for miR-128-3p, preventing mRNA degradation of KLF4	Laminar shear stress	[29]
NFκB	*HOXA-AS2*	Repressing NFκB activation by controlling IκBα degradation and RelA acetylation	Carotid artery atherosclerosis	[37]
	*MALAT1*	Enhancing NFκB activity by sponging miR-150, a NFκB repressor	LPS-induced inflammation, sepsis	[38]
	*TGFB2-OT1*	Sponging miR-3960, miR-4488 and miR-4459	LPS and oxLDL induced inflammation and autophagy	[39]
	*NKILA*	Represses NFκB activity	Released upon inflammation	[33]
GATA2	*LINC00323-003*	Enhancing GATA2 abundance by potential binding translation initiator elF4A3	Regulation of tissue vascularization after hypoxic event	[44]
	*MIR503HG*	Repression of expression of miR-424	Regulation of tissue Vascularization after hypoxic event	[44]
	*GADLOR1 and 2*	GATA2 repressed lncRNAs	Mechanical overload of the heart	[46]
GATA3	*SNHG5*	Potential GATA3 repression by sponging miR-132 and miR-92.	Kawasaki disease	[47]
HIF-1α	*HIF1A-AS2*	Increasing HIF-1α expression by sponging miR-153-3p	Hypoxia	[53]
	*HIF1A-AS2*	Inducing ATF2 expression through recruitment of USF1	Atherosclerosis	[54]
	*HOTAIR*	Scaffolding interaction of LSD1 and HIF-1α	Diabetic retinopathy	[57]
	*MALAT1*	Increasing HIF-1α signaling by sponging miR-19b-3p	Hypoxia	[58]
	*NEAT1*	Securing BBB integrity by sponging HIF-1α-activating miR-135a	Hypoxia	[59]
	*SNHG1*	Securing BBB integrity by sponging miR-18a	Ischemic stroke	[60]
		Sponging miR-140-3p	Myocardial ischemia/reperfusion injury	[61]
	*HypERlnc*	Reduction results in enhanced ER stress through induction of ATF6 activity	Heart failure	[67]
	*GATA2-AS1*	Increasing HIF-1α stability and translation under acute hypoxia	Chronic and acute hypoxia	[62]
SMAD6	*SMANTIS*	Securing SMAD6 expression by enabling BRG1-mediated chromatin remodeling	EC homeostasis	[80]
FOXM1	*MALAT1*	Promoting stability of FOXM1 by sponging miR-320a	EC proliferation	[82]
STAT3	*PVT1*	Preventing STAT3 proteasomal degradation, driving VEGF-driven proangiogenic signaling	Tumor angiogenesis in gastric cancer	[86]
	*SNHG3*	Activating the TWEAK/Fn14/STAT3 pathway and enhancing MMP-2 and MMP-9 expression. Worsening of BBB integrity	Intracerebral hemorrhage	[87]
	*SNHG16*	Enhancing STAT3-mediated proliferation, migration, and invasion by sponging miR-520d-3p	Hemangioma	[88]
	*H19*	Inhibiting STAT3 phosphorylation. Repressing EC senescence, proliferation, inflammatory activation and angiogenic sprouting.	Repressed during aging	[89]
SOX7	*XIST*	Increasing SOX7 abundance by sponging the translational repressor miR-485-3p. Induction of VEGF signaling	Ischemic stroke	[65]
	*HCG18*	Increasing SOX7 abundance by sponging the translational repressor miR-21. Secure barrier integrity by VE-cadherin induction.	Vascular barrier breakdown through ambient particulate matter with an aerodynamic diameter < 2.5 μm	[92]
SOX17	*lncWDR59*	Inducing SOX17 expression to prevent oxidative stress-induced DNA damage	Hyperlipidemia and oxLDL induced maladapted phenotype under disturbed flow	[93]
SOX18	*SMANTIS*	Securing SOX18 expression by enabling BRG1-mediated chromatin remodeling	CKD; mode of action of Dioscin in treatment of CAD	[80,94,95]
